# Integrated Magnetic Resonance Imaging in Muscle-Invasive Bladder Cancer: A Comprehensive Review

**DOI:** 10.7759/cureus.90077

**Published:** 2025-08-14

**Authors:** Nazeer Ibraheem, Mohamed Abosheisha, Ahmed Alemam, Mahmoud Abbas, Momen Abdelglil, Rezuana Tamanna, Mohamed Yasser Elnaggar, Ahmed Swealem, Mohamed Ismaiel

**Affiliations:** 1 Urology, The Royal Wolverhampton NHS Trust, New Cross Hospital, Wolverhampton, GBR; 2 General Surgery, Leicester Royal Infirmary Hospital, University Hospitals of Leicester, Leicester, GBR; 3 General Surgery, Leicester Royal Infirmary Hospital, Leicester, GBR; 4 Pediatric Surgery, Mansoura University Children's Hospital, Mansoura, EGY; 5 General Surgery, Watford General Hospital, Watford, GBR; 6 Head and Neck Surgery, Mansoura University Children's Hospital, Mansoura, EGY; 7 Orthopedics, North Bristol NHS Trust, Bristol, GBR; 8 General Surgery, University Hospital Limerick, Limerick, IRL

**Keywords:** bladder cancer, bladder cancer imaging, mri, mri-urography, multiparametric mri (mpmri), muscle-invasive bladder cancer (mibc), radiological evaluation, staging of bladder cancer, translational uro-oncology, tumor detection

## Abstract

The integration of multiparametric MRI (mpMRI) into the diagnosis and treatment of muscle-invasive bladder cancer (MIBC) has significantly enhanced decision-making. This review highlights advancements in mpMRI, including its applications in tumor grading, treatment response assessment, and post-treatment surveillance. The use of artificial intelligence further enhances predictive capabilities. However, challenges remain, particularly regarding accessibility and interobserver variability. mpMRI represents a pivotal, noninvasive tool that supports precision medicine and bladder-preserving strategies in MIBC care.

## Introduction and background

Muscle-invasive bladder cancer (MIBC) is a major health problem. Bladder cancer has an estimated incidence rate of 9.5 per 100,000 men and 2.4 per 100,000 women, making it the seventh most common cancer in men and the 10th most common in women. According to recent research, it also accounts for approximately 83,190 new cases and 16,840 deaths in the US in 2024, with muscle-invasive disease accounting for about 25% of newly diagnosed patients [1,2].

Bladder cancer incidence varies by region, as developed countries have the highest incidence, especially in Southern and Western Europe, where smoking and industrial exposures raise risk [1].

Awareness of the differences between non-muscle-invasive bladder cancer (NMIBC) and MIBC is important due to the distinct differences in disease progression and management. While about 75% of newly diagnosed bladder cancer cases are confined to the mucosa or submucosa, a notable proportion may present as muscle-invasive at diagnosis [3,4].

MIBC carries a poor prognosis when compared to NMIBC, reflecting its aggressive nature and high incidence of recurrence after treatment. Systemic recurrence rates following cystectomy remain substantial, ranging from 20% for stage pT2 to more than 50% for stage pT4 [2].

The need for accurate preoperative assessment and improved staging is underscored by the clinical challenges in managing the disease, including risks of understaging with transurethral resection and the limitations of conventional imaging modalities. These factors highlight the importance of ongoing improvement in diagnostic techniques and integrated imaging, such as MRI [5]. This review aims to examine the current role, diagnostic accuracy, and future directions of MRI in the evaluation and management of MIBC.

## Review

Methodology

The methodology for this review was designed to ensure comprehensive application of the latest research and clinical advancements in the usage of MRI for MIBC. A systematic literature search was conducted using the databases PubMed, Scopus, and Web of Science. Search terms included "muscle-invasive bladder cancer", "multiparametric MRI", "bladder cancer imaging", "VI-RADS", "AI in bladder MRI", and related synonyms. Boolean operators were employed to combine terms to achieve maximum sensitivity in capturing relevant literature. The search was restricted to studies published between January 2020 and July 2025 to ensure inclusion of recent developments and emerging trends.

Inclusion criteria encompassed peer-reviewed original articles, systematic reviews, and meta-analyses focused on MRI in MIBC. Studies evaluating technical advances in MRI, VI-RADS (vesical imaging reporting and data system) validation, artificial intelligence (AI) integration, and the clinical impact of imaging were included. Only English-language publications with accessible full texts were considered. Exclusion criteria included case reports, editorials, conference abstracts, and non-English publications, as well as studies that focused exclusively on NMIBC.

The study selection process involved two independent reviewers who screened titles and abstracts for relevance, followed by full-text assessment of eligible studies. Data extracted from each study included study design, patient population, MRI protocol details, diagnostic performance metrics, integration of advanced imaging or AI, and reported clinical outcomes.

Although no formal risk-of-bias method was used, studies were critically appraised based on their study design, methodological type, and relevance to the objective. Special attention was given to studies that highlighted the integration of MRI in MIBC.

MRI protocol optimization in MIBC

Recent studies highlight the importance of multiparametric MRI (mpMRI) protocols in improving diagnostic accuracy for MIBC. These protocols include high-resolution T2-weighted imaging, diffusion-weighted imaging (DWI), and dynamic contrast-enhanced (DCE) sequences, with 3.0T MRI preferred for better image quality. Proper patient preparation, such as optimal bladder filling, is also crucial. Standardization, particularly through the use of the VI-RADS system, enhances inter-reader reliability and diagnostic consistency. Adherence to these protocols can improve the accuracy and precision, aiding in treatment planning and supporting bladder-preserving approaches [6-8].

The consensus among researchers is that an optimized mpMRI protocol for MIBC should include T2-weighted sequences for anatomical detail, DWI for tumor defining, and DCE-MRI for assessing perfusion. DWI and DCE imaging are especially important for differentiating tumor tissue from surrounding parts and for identifying muscle or perivesical fat invasion, which impacts T-staging. Notably, combining these sequences in a multiparametric approach yields higher diagnostic accuracy than a single sequence alone, with reported sensitivities and specificities for differentiating non-muscle-invasive from muscle-invasive disease exceeding 90% in well-conducted studies [7,9]. The BladderPath trial and other multicenter validations confirm that the routine integration of mpMRI, adhering to VI-RADS and protocol compliance, is now feasible in clinical practice, with high rates of protocol adherence and diagnostic yield [6].

Development and validation of VI-RADS

The VI-RADS was used in 2018 to provide a standardized protocol for bladder cancer evaluation using mpMRI. It aimed to improve the detection and staging of MIBC by reducing interobserver variability and establishing a structured reporting system. VI-RADS uses a five-point scale to categorize the likelihood of muscle invasion, integrating T2-weighted, DWI, and DCE imaging sequences within a unified reporting framework [10]. The original development and validation studies highlighted a strong correlation between VI-RADS scoring and histopathologic diagnosis of muscle invasion, marking a significant advancement over traditional imaging and cystoscopy approaches [11].

Diagnostic Accuracy and Interobserver Agreement

Numerous studies have demonstrated that VI-RADS has high diagnostic accuracy in differentiating MIBC from NMIBC. Meta-analyses and systematic reviews have pooled data from thousands of patients, reporting sensitivity values ranging from 83% to 97% and specificity values between 74% and 97% [12,13]. The cutoff value of ≥3 is widely used to optimize the balance between sensitivity and specificity. Notably, VI-RADS also exhibits strong interobserver agreement, with pooled κ coefficients consistently exceeding 0.80, underscoring its reliability across varying levels of reader experience [13,14].

Clinical Implementation and Applications

**Figure 1:**
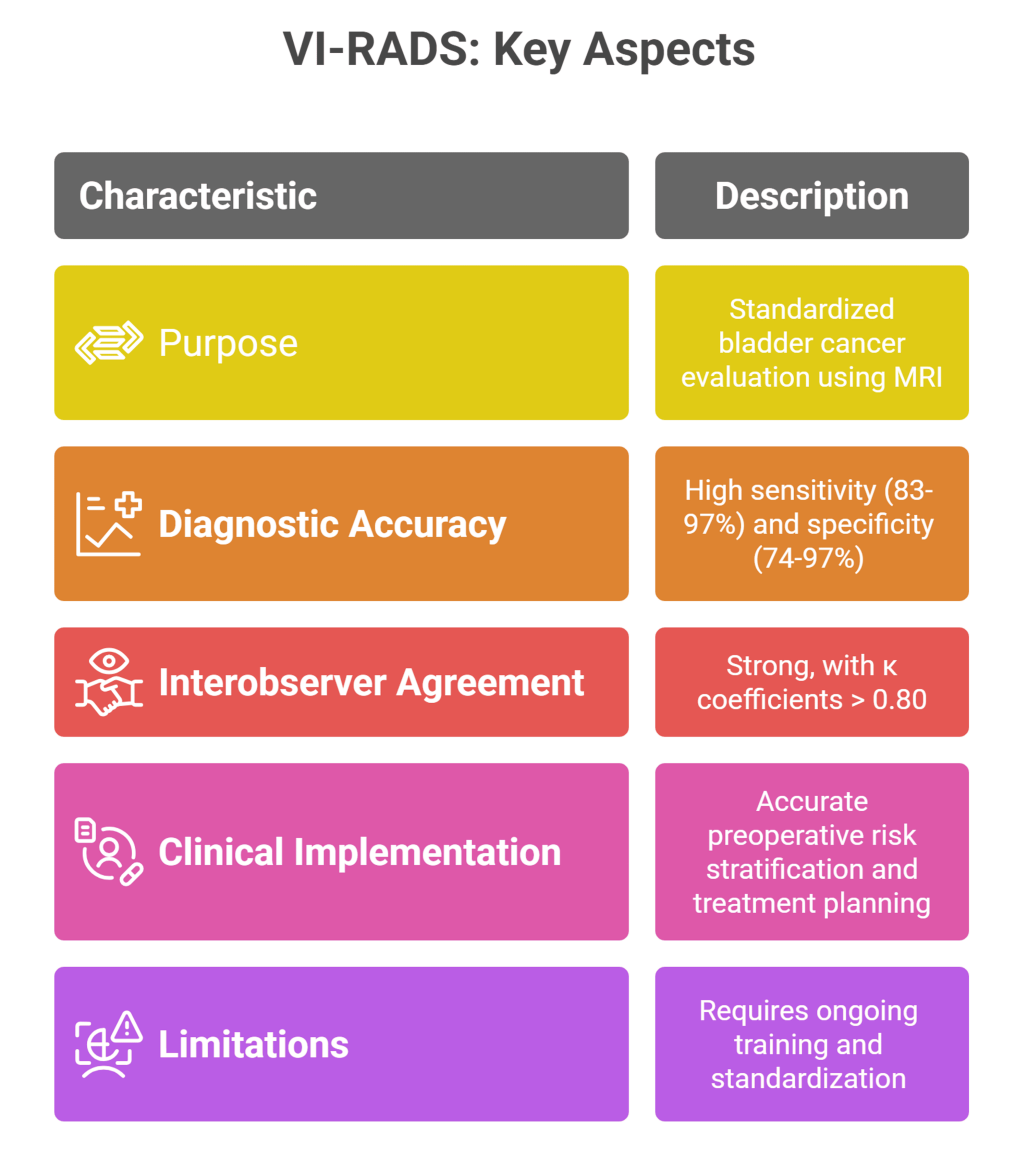
Development and Validation of Vesical Imaging Reporting and Data System (VI-RADS). VI-RADS: Vesical Imaging Reporting and Data System Image Credit: Momen Abdelglil Source: [10–14]

The integration of VI-RADS into clinical practice can provide more accurate preoperative risk stratification, aiding in the selection of candidates for radical cystectomy or neoadjuvant chemotherapy (NAC) [10,15]. Several studies have explored how VI-RADS scoring may help determine the necessity of repeat transurethral resection, especially in cases where muscle invasion is uncertain after initial resection [16]. Additionally, VI-RADS has shown good utility in personalizing treatment plans, monitoring response to neoadjuvant therapies, and guiding surveillance types for recurrent disease. Despite these advances, research highlights the need for ongoing training, standardization, and multicenter validation to optimize its effectiveness and incorporate it into bladder cancer care protocols (Figure 1) [12,14].

Diagnostic performance of multiparametric MRI

mpMRI has emerged as a highly effective tool for the primary staging of MIBC [9,17].

Tumor Grading and Histological Prediction

Beyond local staging, mpMRI offers significant power in evaluating tumor grade and predicting histological types. Quantitative MRI metrics, like the apparent diffusion coefficient (ADC), are inversely correlated with tumor grade. Lower ADC values are associated with higher-grade and more aggressive tumors, supporting MRI's use as a noninvasive imaging biomarker for aggressiveness [9]. Large radiomics studies have developed MRI-based clinical-radiomics models to predict grade and variant, achieving AUCs between 0.73 and 0.93, suggesting relatively high performance for noninvasive grade prediction [9,17].

Preoperative Risk Stratification

Multicenter evidence indicates that VI-RADS scoring, combined with clinical factors such as hematuria and tumor size, is an independent predictor of muscle invasion, improving the prediction of ≥T2 disease before surgery. This combined approach may help identify high-risk patients who may benefit from expedited or more aggressive therapy, potentially leading to altered patient management types [18].

Treatment response assessment

Quantitative MRI features, including radiomics analysis, aid in predicting short-term response to neoadjuvant treatments, disease-free survival, and long-term outcomes. The integration of MRI findings with clinical, molecular, and pathological data offers a good evidence-based approach for risk-adapted patient management in MIBC [19,20].

NAC Monitoring

mpMRI, particularly DWI and VI-RADS, is increasingly validated for assessing MIBC response to NAC. These imaging tools provide a noninvasive means to predict pathological complete response (pCR), with specific MRI-derived systems, such as changes in ADC values, correlating closely to chemotherapy effectiveness and patient prognosis [20,21].

MRI in Immunotherapy Response

Complete and partial responders to neoadjuvant immunochemotherapy can be distinguished non-invasively using MRI using sophisticated techniques, such as amide proton transfer-weighted imaging. MRI's use in therapy evaluation and individualized treatment planning for patients with bladder cancer is supported by post-treatment decreases in amide proton transfer-weighted values and increases in ADC values, which are indicators of positive response [22].

AI and machine learning integration in MRI for MIBC

AI and machine learning have rapidly advanced the MRI-based assessment of MIBC. Recent studies showed the superiority of deep learning (DL) models, such as vision transformers and convolutional neural networks, in diagnosing MIBC when compared to traditional radiologists, with area under the curve (AUC) values frequently exceeding 0.85 and even outperforming experienced radiologists in accuracy and speed [23]. DL models can also complement established systems, such as VI-RADS, especially in challenging cases [24,25].

**Figure 2:**
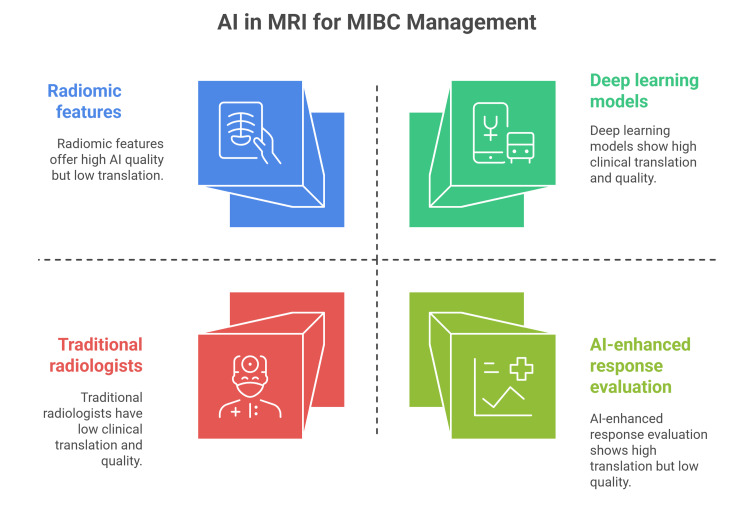
Artificial Intelligence and Machine Learning Integration in MRI for Muscle-Invasive Bladder Cancer. MIBC: Muscle-Invasive Bladder Cancer Image Credit: Momen Abdelglil Source: [23-30]

Machine learning approaches utilizing radiomic features from multiparametric MRI provide improved preoperative staging and assessment of histologic variants, offering noninvasive, cost-effective decision-making methods [26,27]. Quantitative imaging biomarkers and radiomics, enhanced by AI, can improve standard response evaluation criteria, providing earlier and more precise treatment response [28]. However, critical reviews emphasize that despite promising accuracies and sensitivities, the quality and transparency of many AI studies remain limited, often demonstrating a high risk of bias [29]. Therefore, many multicenter validations, standardized data protocols, and ethical considerations, such as bias mitigation and explainability, are crucial for the clinical translation of AI-powered MRI tools in MIBC management (Figure 2) [29,30].

Surveillance and follow-up applications

Recent research proves the growing role of mpMRI in the surveillance and follow-up of MIBC, aiming to increase diagnostic accuracy and reduce unnecessary interventions. Studies demonstrate that mpMRI is valuable in differentiating post-treatment inflammatory changes from tumor recurrence, especially after intravesical therapy or transurethral resection (TURBT). For example, MRI has shown superior specificity and negative predictive value compared to cystoscopy, particularly in cases where cystoscopic findings are equivocal after treatment [31,32]. This results in fewer unnecessary repeat resections and improved disease outcomes, notably in patients evaluated for flat lesions, where cystoscopy is often inconclusive [32]. Additionally, DWI and advanced MRI protocols can better differentiate between inflammatory responses and malignant recurrence, potentially transforming the conventional follow-up strategies [8].

Several prospective studies have also shown that MRI-based surveillance is feasible and effective, allowing the earlier detection of recurrence [31,33]. The integration of standardized assessment systems, such as VI-RADS, further enhances the reliability of follow-up imaging results, particularly for monitoring patients undergoing bladder-preserving therapies or neoadjuvant treatments [8,34]. Despite these advances, it is important to note that MRI interpretation remains operator-dependent and may benefit from additional training [8]. Overall, the use of mpMRI in post-treatment surveillance is reshaping follow-up protocols in MIBC and holds promise for individualized, risk-adapted patient care [34,35].

Limitations and challenges

Despite the promising role of mpMRI in evaluating MIBC, several limitations and challenges persist. One technical limitation lies in the accuracy of lymph node staging, where mpMRI shows relatively poor accuracy and performance, impacting comprehensive cancer assessment. Moreover, issues such as MRI spatial resolution constraints and artifacts can affect image quality. The imaging of large or multifocal tumors is particularly challenging, contributing to risks of understaging, which may delay appropriate treatments. Additionally, interobserver variability and the requirement for high operator expertise may be significant barriers to consistent interpretation, particularly when MRI scoring systems such as VI-RADS are applied outside specialized academic centers [9,36].

The need for contrast agents in DCE sequences raises concerns about patient safety, potential side effects, and limits applicability in certain patient populations. Furthermore, the accessibility of MRI technology, cost considerations, and potential delays in treatment initiation due to imaging schedules present practical hurdles to the widespread implementation of mpMRI in routine clinical pathways [9,37].

**Figure 3:**
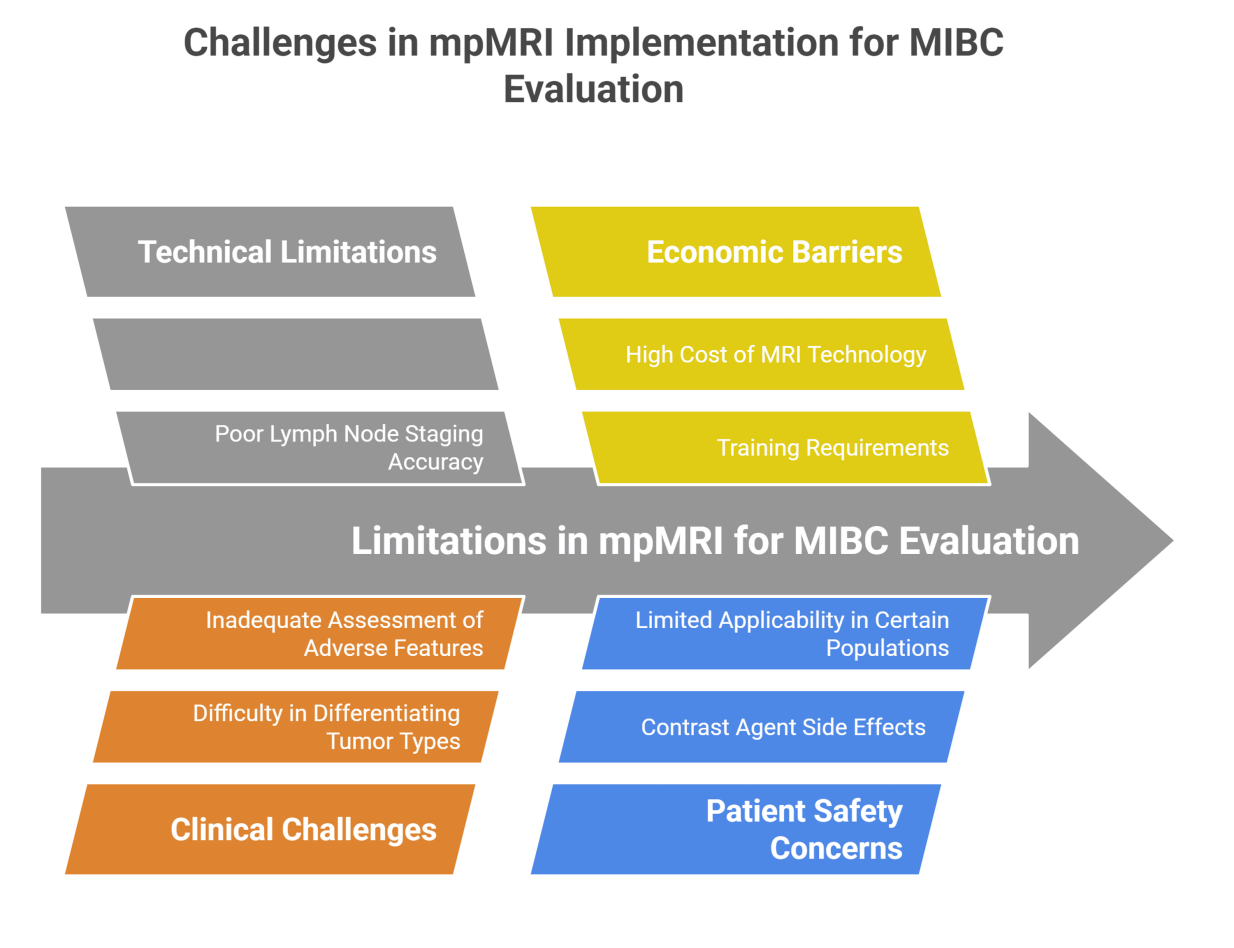
Limitations and Challenges in mpMRI for MIBC. MIBC: Muscle-Invasive Bladder Cancer; mpMRI: Multiparametric MRI Image Credit: Momen Abdelglil Source: [34-38]

Clinically, the limitations of mpMRI in accurately differentiating muscle-invasive from non-muscle-invasive tumors impact its diagnostic reliability. Although the high sensitivity and specificity of the mpMRI have been proven, overstaging and understaging of tumors remain concerns, especially in complex cases involving tumor heterogeneity and variant histologies that the mpMRI cannot reliably detect. Adverse pathological features such as carcinoma in situ, lymphovascular invasion, and ureteral infiltration are also inadequately assessed through current mpMRI protocols. Finally, the economic burden of mpMRI and the associated training requirements could limit its adoption, particularly in non-academic or resource-limited settings. As such, while mpMRI and standardized systems like VI-RADS have improved preoperative staging and risk stratification, they should be integrated judiciously with histopathological and clinical data to guide patient management until further validation studies and technological advancements address these challenges (Figure 3) [9,34,38].

Future perspectives and emerging technologies in bladder MRI

Advancements in bladder MRI are rapidly changing the landscape of diagnosis, risk stratification, and therapy monitoring for MIBC. Recent studies have emphasized the refinement of MRI-based scoring systems, such as VI-RADS, and the development of NAC-VI-RADS [38]. Quantitative MRI parameters, such as ADC values and radiomics features, have opened new avenues for risk assessment and prognostication, allowing clinicians to perform more personalized staging and monitoring. Radiomics, in particular, offers a promising non-invasive method for predicting response to NAC, with signatures derived from pre-treatment imaging showing potential as predictive biomarkers for favorable pathological outcomes, although larger studies are still needed to validate these models [38,39].

**Figure 4:**
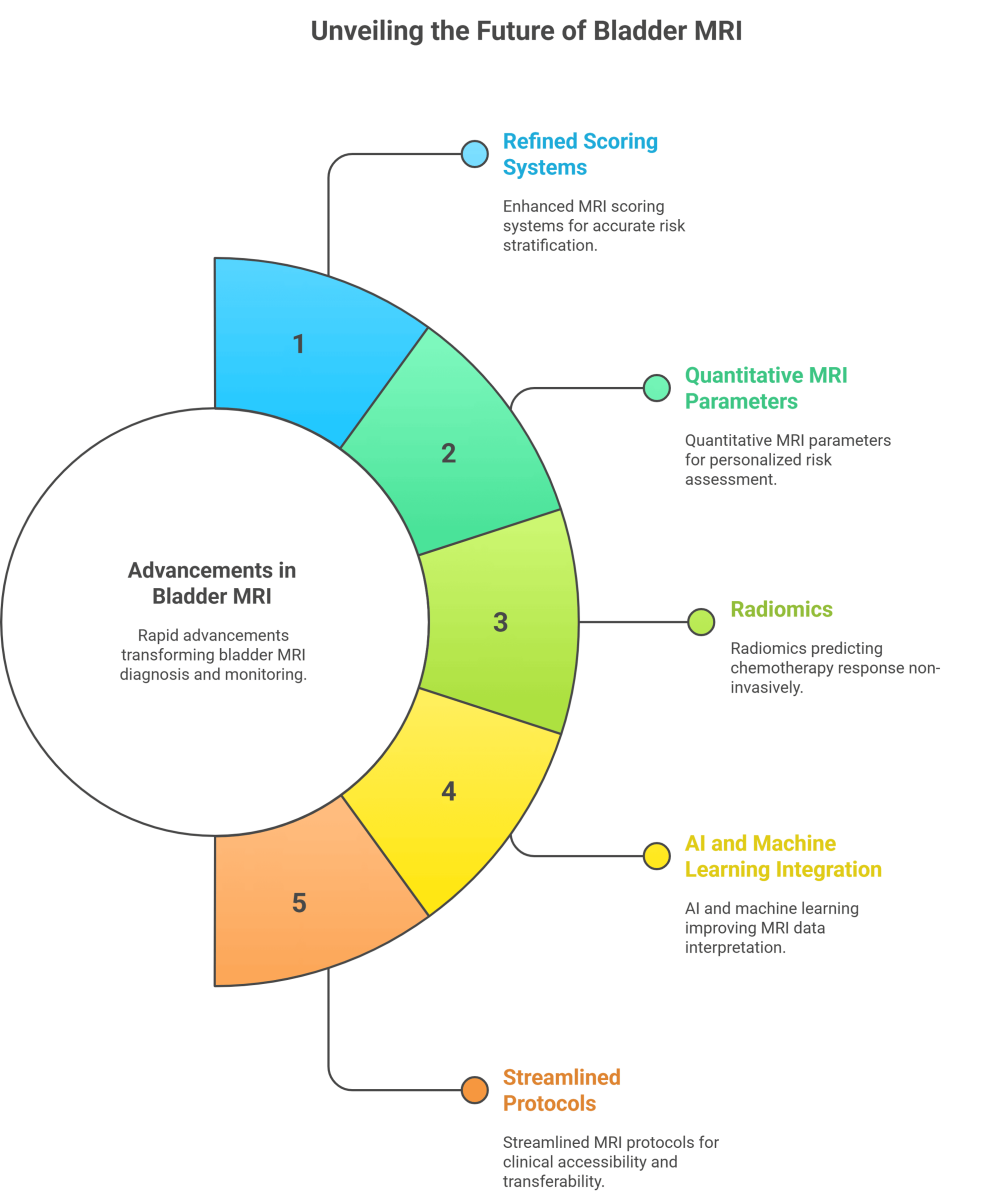
Future Perspectives and Emerging Technologies in Bladder MRI Image credit: Momen Abdelglil Source: [38,39]

Researchers are increasingly focused on integrating AI and machine learning into the interpretation of MRI data, enhancing diagnostic power and enabling more refined predictions of muscle invasion and treatment response [38]. By supplementing conventional anatomical imaging with quantitative radiomic features and machine learning, the accuracy of preoperative staging, risk assessment, and prognosis has markedly improved, paving the way for future applications in precision medicine. Moreover, there is ongoing work to streamline MRI protocols and scoring systems, making these tools more accessible and transferable to clinical practice, while addressing the variations in technique and interpretation that remain as challenges (Figure 4) [38,39].

## Conclusions

mpMRI, supported by VI-RADS and AI-driven analytics, has emerged as a good tool in the diagnosis, staging, and follow-up of MIBC. Its ability to enhance diagnostic accuracy, guide personalized treatment decisions, and reduce unnecessary interventions represents a notable and important advancement toward precision oncology in urothelial malignancies.

However, widespread adoption remains limited by cost, accessibility, operator dependence, and technical constraints, particularly in lymph node staging and variant histology detection. Continued multicenter validation, standardization of imaging protocols, and integration with histopathological and molecular data will be essential to maximize its clinical impact and ensure equitable implementation.
